# Supplementary figures and images for: Targeting CA-125 Transcription by Development of a Conditionally Replicative Adenovirus for Ovarian Cancer Treatment
Source: Cancers (Basel). 2021 Aug 24;13(17):4265. doi: 10.3390/cancers13174265 (PMC8428227; doi:10.3390/cancers13174265)

E1A

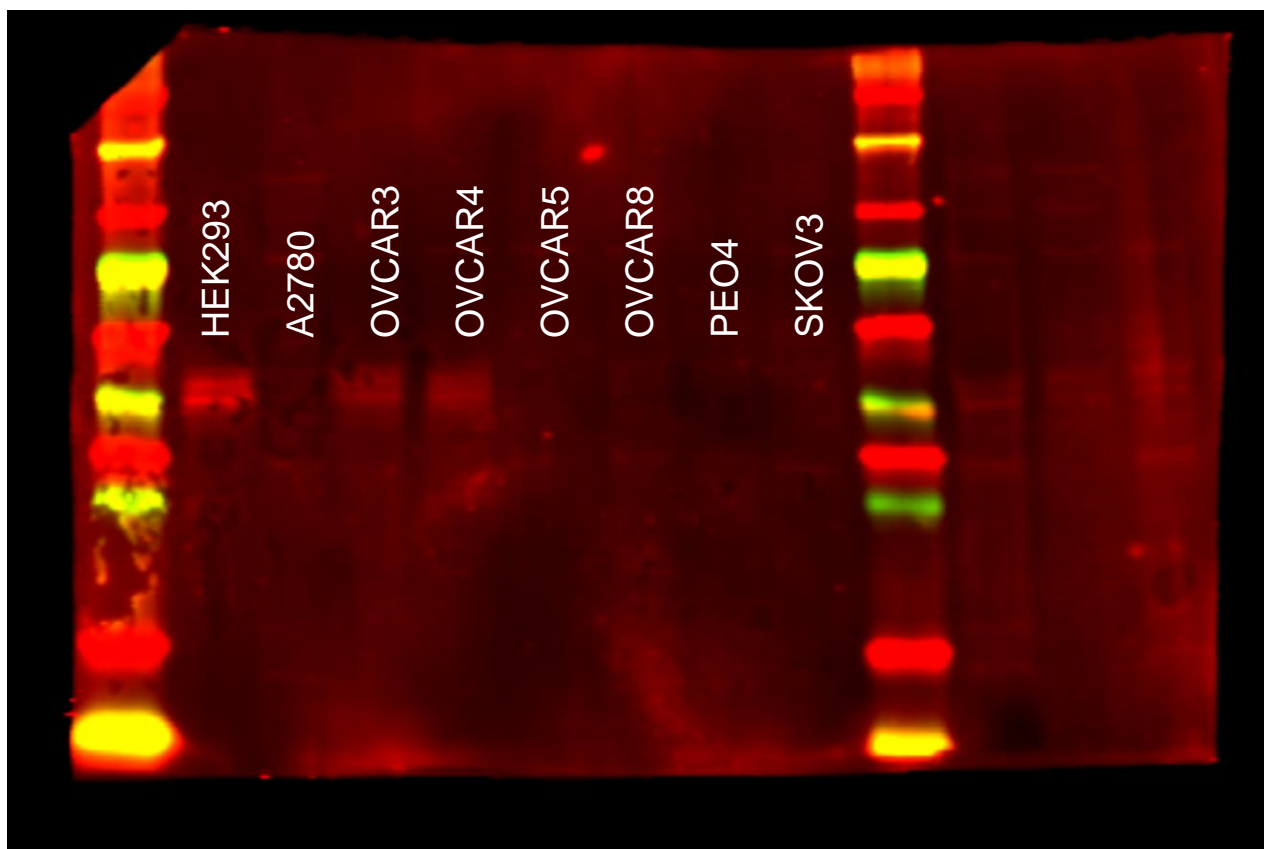

$\beta$ -actin

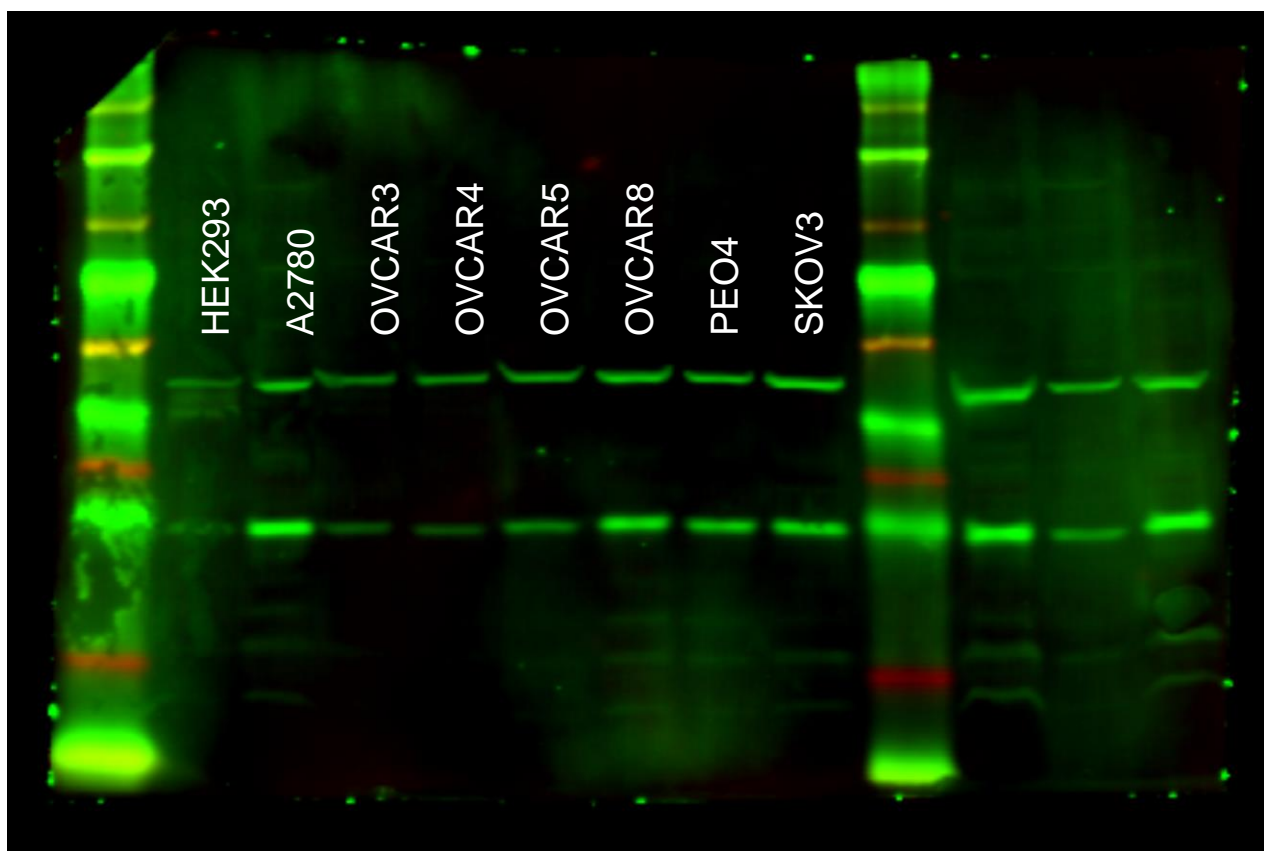

Supplement: Supplementary file 1 [file cancers-13-04265-s001.zip › cancers-1310278-supplementary.pdf]
